# Book Review

**Published:** 1932

**Authors:** 


### Reviews of Books

**Health and Hygiene in England from Roman to Victorian Times.** By J. A. Delmege, O.B.E., M.R.C.S. Pp. xiv., 234. Illustrated. London: William Heinemann Ltd. 1931. Price 21s.—This book gives a brief but most interesting history of the changes in public health and of the growth of the hygienic art in this country for 1,800 years. The story of the epidemics here, and of the food, housing, clothing, and the industrial conditions of the people at different epochs is a marvel of good condensation and, indeed, of accuracy, when we consider the vast accumulation of facts here collected, though, of course, there is room for divergent opinions as to the inferences to be drawn from some of them. It is intended chiefly for the lay reader, and, as Sir Thomas Legge in a foreword says, it "can be read with pleasure and unfailing interest from end to end by everyone." Yet the medical scientist, too, cannot fail to find much of value in these records of the course of disease in the past. Thus, to take an instance, we learn some of the causes of the huge mortality of infants in former times. Besides the absence of air, light, and warmth in the houses are, for those who could not be breast fed, the absence of any pure cows' milk or infants' food, and the constant presence of infectious fevers of various kinds and overcrowding in the towns. This is worked out in detail with illustrative facts. The great work of Howard, Chadwick, Shaftesbury, Owen and Nightingale fill many delightful pages. We think it might be made clear that most almshouses, unlike the hospitals, survived the Dissolution under the Tudors, and we suggest that very few poorhouses under the Act of Elizabeth were set up till Carey's Act of 1696 allowed parishes to form Unions. In the next forty years no less than 115 were set up and the progress went on steadily. The book is beautifully printed and illustrated, but the paper is very heavy for a book of this size.

**Sixty Centuries of Health and Physick.** By S. G. B. Stubbs and E. W. Bligh. Pp. xvi., 253. Illustrated. London: Sampson Low, Marston & Co. Ltd. Price 15s.—In his Introduction to this history of medicine Sir Humphry Rolleston has summarized its point of view by the remark: "In tracing the evolution of 'physick' from primitive magic to modern medicine the authors have successfully brought out the importance of the preventive idea which should permeate the teaching, practice and research of the medicine of the future." The authors have indeed achieved a notable success in tracing out the progress of ideas rather than cataloguing the biographies of individual scientists and collecting historical incidents. Their method has been "to seize the prevalent ideas of each age of medicine," so as to give the reader a clear conception of the growth of medical science and particularly of hygiene. The result of their labours enables them to present a continuous story of surpassing interest, documented by numerous quotations from out-of-the-way sources and aptly furnished with beautiful illustrations.

**A Theory of the Formation of Animals.** By W. T. HILLIER, M.R.C.S. Pp. viii., 166. Illustrated. Bristol: John Wright & Sons Ltd. 1932. Price 8s. 6d.—This book, attractive in style and in title, presents a theory of the dual in our constitution. Dr. Hillier takes the blastula for the type of all metazoa; antecedent to the conjugation of ovum and sperm have been the collision and collusion of blastula-like organisms. These are dissevered in the gastrula so that the cellular descents from each are thenceforward separate. Serial and segmented organs mark the intercalation of asexual phases of growth. Cells find their place in the body's finished symmetry, under the dominance of an "active meridian." For proof the author goes to Polygordius, a minute annelid of German seas, within whose primitive lines can be easily traced the tendencies of growth in embryo. The argument is carried to vertebrates in an elaborate study of the anatomy of the herring, bony homologies of which are brought out in some beautiful illustrations.

**Modern Medical Treatment.** 2 volumes. By E. BELLINGHAM-SMITH, M.D., F.R.C.P., and A. FEILING, M.D., F.R.C.P. Pp. xvii., 701; vii., 705. London: Cassell & Co. Ltd. 1931. Price 30s.—We should expect this work to attain a very high standard, and we are certainly not disappointed. There is here no mere statement of numerous therapeutic methods which have been copied from one book to another to such an extent that the reader turns wearily to some foreign work for further information. On the contrary, every section of the book, without exception, is so well written, so well balanced, so free from fad, and so modern in outlook, that we are bound to feel that it must supply a much-felt want. At the commencement of each chapter the rationale of treatment is outlined. From the point of view of the student, this must form one of the most valuable features of the book. Further, where necessary the writers have not failed to indicate the various ætiological factors upon which the method of treatment depends. Thus they state that "it is unfortunate that the term sciatica has been used loosely to designate almost any kind of pain in the buttock, hip or thigh." They then proceed to discuss the various causes of sciatic pain, and show clearly how treatment must depend upon the diagnosis. Again, to physician and surgeon alike, might be commended the measured discussion on hyperthyroidism, Graves's disease and toxic goitre. It is to be regretted, however, that the treatment of the latter condition is not really outlined. It is difficult to criticize a work of such uniform excellence. A few suggestions, however, may not be untoward. Thrombosis of the coronary arteries is surely worthy of more discussion? Again, in considering the treatment of arthritis associated with chronic infection of the prostate no mention is made of the application of diathermy to the focus of infection. Recurrent aphthæ in the mouth may be very difficult to treat, and it is well that this affection is briefly considered, though it is to be regretted that no mention is made of a treatment which is of real value, namely the application of colloidal mercurochrome. Finally, scarlatinal antitoxin would be a better phrase than "antiscarlatinal antitoxin." These and others, however, are minor details which detract but little from a work which may be said to be a worthy product of the London schools of medicine.

**The Thyroid and Manganese Treatment.** By H. W. NOTT, M.R.C.S. Pp. xv., 265. London: William Heinemann Ltd. 1931. Price 7s. 6d.—In this volume the author describes in detail the steps by which he was led to regard the "Thyroid and Manganese Treatment" as a cure for the majority of diseases. The treatment consists of the administration of thyroid extract by mouth, together with rectal injections of a solution of potassium permanganate. In certain cases, instead of the rectal injection, potassium permanganate may be given orally in a cachet, but as a rule the results are not nearly so satisfactory. It is claimed that this treatment will cure all acute infections, including pneumonia and meningococcal meningitis, and such diverse diseases as diabetes mellitus, gastric ulcer, toxæmias of pregnancy, and varicose ulcers, to mention only a few. Most of the diseases met with in general practice tend to improve whatever treatment is adopted, and it is easy for an observer sufficiently enthusiastic about any special form of therapy to ascribe this improvement to his treatment and not to nature.

**Motives and Mechanisms of the Mind.** By E. G. HOWE, M.B. Pp. xiii., 260. Illustrated. London: The *Lancet.* 1931. Price 10s. 6d.—Many books have been written on psychopathology and psychotherapy in the last ten years, and a new volume on the same subject must necessarily be more critically approached than would have been the case a decade ago. These twelve lectures were delivered to a post-graduate course, and they merit the highest praise, for they sum up modern thought on the subject in a clear and attractive style. If the reader expects some new and original contribution he will be disappointed, but the author is not to blame, for he has not set out to give this. For postgraduate teaching also a correct proportion has been maintained, since eleven and a half chapters are devoted to the "physiology" (*i.e.* psychology) and pathology of the subject and a half to treatment. The author is critical of all "theories," but applies what we have learnt from the great analytic schools with sound common sense. He traces the development of our unconscious and conscious motives, and shows how they influence our behaviour both individually and collectively. If the practitioner troubles to study this book thoroughly, he will neither scoff at what he does not understand nor rush in where the angels fear to tread in an access of enthusiasm for a subject which he thinks others do not understand.

**Rheumatoid Arthritis and its Treatment.** By VINCENT COATES, M.C., M.D., M.R.C.P., and L. DELICATI, L.M.S.S.A. Pp. xv., 114. Illustrated. London: H. K. Lewis & Co. Ltd. 1931. Price 6s.—This small monograph consists, as the sub-title suggests, of "Studies from the Royal Mineral Water Hospital, Bath." Though somewhat brief, it should prove useful by showing that something besides physic may be given for arthritis. The chapter on joint treatment is good as far as it goes. Stress is rightly made on the prevention of contractures. Perhaps more might have been said with regard to exercises, though it is good to find that breathing exercises are not ignored. The focal infection bogey is put in its proper place. Some of the illustrations might be improved upon.

**Studies in the Photo-activity and Therapy of the Tungsten-Titanium Arc.** By J. BURDON-COOPER, M.D., F.R.C.S., and A. ROBERTS, F.R.C.S. Pp. 85. Illustrated. Bristol: John Wright & Sons Ltd. 1931. Price 10s.—The book is a record of experimental and clinical work in actinotherapy with an open arc lamp, using special electrodes of an alloy of tungsten, titanium and chromium. The authors claim that these electrodes give a continuous spectrum, and that the addition of titanium intensifies those regions where the tungsten spectrum is weak, while the resulting spectrum more nearly resembles that of natural sunshine. The book is divided into two parts: (1) experimental; (2) clinical. The experimental part of the book is very convincing, dealing as it does with the penetration of the various portions of the spectrum and the absorption value of the rays. The experiments are ably demonstrated by the camera, and the photographs are of admirable quality, clearly showing the results arrived at. The authors are unable to explain all the phenomena found and frankly consider that the experiments are incomplete. Part II. contains chapters on the Crombie lamps and their application, and while the authors do not claim a final definite conclusion, the clinical details are carefully worked out and illustrative cases submitted, although the results would not appear to be markedly superior to ultra-violet radiation obtained from other sources.

**Minor Surgery.** By L. R. FIFIELD, F.R.C.S. Pp. viii., 439. 281 illustrations. London: H. K. Lewis & Co. Ltd. 1931. Price 12s. 6d.—The reviewer had the pleasure and privilege of reviewing the first edition of this work, about seven years ago. He found few faults and gave much praise, and in reading the second edition he sees no reason to withdraw any complimentary remarks, indeed it shows some improvement on the former. There is some slight addition in the section on the examination of a patient, and it includes a short account of the more common ear conditions not described in the first edition. The book should be in the hands of every student commencing his surgical work, and even those of more eminent attainment might well appreciate its sphere of usefulness. The author and reviser have omitted to include procedures and apparatus whose use has been discarded for the past forty years, and in this respect there is a distinct difference between this and other volumes of similar scope. It is a sad catastrophe which has robbed the medical profession of any prospect of further works from the pen of so prominent a man and so promising a writer as the late author.

**Diagnosis in Joint Disease.** By N. ALLISON, M.D., F.A.C.S., and R. K. GHORMLEY, M.D. Pp. xi., 196. Illustrated. London: Oxford University Press. 1931. Price 52s. 6d.—This monograph represents six years' intensive study by two experienced observers of various joint diseases, 289 cases actually forming the basis of the thesis. It is an essentially practical book, dealing with theories of causation and etiology only so far as these are necessary to elucidate or justify classification. All the more important diseases, *e.g.* tuberculosis, pyogenic infection, traumatic arthritis, etc., are dealt with by means of a series of clearly-described case reports, illustrated by X-rays taken at different stages of the disease and by several microphotographs of tissue removed at operation. For purposes of reference these case reports, and especially the correlation of the pathological with the clinical aspects, form a most valuable storehouse of information. The general production of the book and of the illustrations is excellent, but we think it unfortunate that coloured photography has been used for some of the coloured plates, because such reproductions do not give the necessary characteristic definition.

**Emergency Surgery.** Volume 2. By HAMILTON BAILEY, F.R.C.S. Pp. xvii., 415. 430 illustrations. Bristol: John Wright & Sons Ltd. 1931. Price 25s.—This volume is a worthy companion to its fellow on abdominal emergencies. The style is pithy and pleasing, and all the post-war additions to surgery of head, chest and limbs are included. The illustrations are admirable in quality and quantity. The course and treatment of septic infection of the hand and fingers are dealt with in a first-rate form after the teaching of Kanavel. Concise and correct advice is given on coping with injuries to bone and soft parts of the limbs, but, to be quite up to date, there should be mention of Kirschner's transfixion wire for extension. Blood transfusion has the praise and place that it deserves. It is good to see Bristol represented by the Ear, Nose and Throat Section contributed by Mr. Eric Watson-Williams, and it is pleasant to see Mr. Chitty's successful iodine treatment of actinomycosis recognized, even though the text-book is one of emergency surgery.

**Technique and Results of Grafting Skin.** By H. K. CHRISTIE, M.S., F.R.C.S. Pp. xii., 67. Illustrated. London: H. K. Lewis & Co., Ltd. 1930. Price 7s. 6d.—The title of this small volume aptly describes its contents, and little can be added to enlighten the prospective reader. The writer deals with the recognized types of skin graft, their application and chief indications, concisely and without any elaboration, bias, padding, or phrases of laudatory comment on his own achievement. The outstanding feature is the apparent success of large Wolff grafts. A short history of a limited number of cases is given. The book should be of value to those who contemplate the undertaking of plastic surgery without the backing of practical experience.

**The Genesis of Cancer.** By W. SAMPSON HANDLEY, M.S., F.R.C.S. Pp. xix., 258. Illustrated. London: Kegan Paul, Trench, Trubner & Co., Ltd. 1931. Price 21s.—Mr. Sampson Handley's main thesis is that cancer is due to the effects on the epithelium of long-standing lymphatic obstruction. He begins by showing the true character of the lymphatics of the skin, which is given obscurely or erroneously in the text-books. They commence as blind, tube-like vessels, one in the centre of each papilla of the skin, and pour lymph into collecting vessels in the dermis, which drain an area of skin about half an inch across, and show little or no lateral anastomosis with other areas. Plunging deep, these vessels enter the general freely-anastomosing plexus in the deep fascia, from which main lymphatic trunks carry the fluid to the nearest lymphatic glands. When the lymphatics of the papillæ and dermis are obstructed by inflammatory overgrowth of their living endothelium, the epithelium of the skin grows upwards and downwards, and a papilloma results. Sections of papilloma show the obstructed lymph-vessels in the papillæ and in the dermis. Chronic obstructive lymphangitis of characteristic "stag-antler" pattern can be demonstrated in lupus, in warts, in paraffin dermatitis, in the irritation produced by rubbing tar into a mouse's skin, and in X-ray or radium dermatitis, all of which are precancerous conditions. In pigmented moles the lymph-vessels in the papillæ of the skin are dilated and empty; here, too, there is a tendency to malignant degeneration. The author thinks that cancers of the stomach and intestine usually arise in adenomatous polypi, and that these are due to lymph-stasis, but "the question needs further investigation." Cancer of the breast, as Sir Lenthal Cheatle and others have shown, begins as a proliferation of the epithelium lining the ducts of one or other quadrant of the mammary gland. The epithelial overgrowth is papillomatous or "laciform" in character. Cheatle gives no clear explanation as to why this overgrowth occurs. Sampson Handley points out that in cases of chronic mastitis

(=" proemial breast " of Cheatle) the ducts are surrounded by dilated lymph-spaces, and, at a later stage, by hyaline fibrous tissue, which he believes is the result of inflammation and obstruction of the lymphatic vessels. The sequence of events is as follows: infection of the periductal lymphatics by germs entering at the nipple; lymphatic inflammation and obstruction; wart-formation in the ducts; cancer. It is a fascinating book, and the theory is, at least, entitled to respect. One wonders if it is not *too* simple. In biology simple explanations are apt to prove fallacious.

**Wayfaring in a Splint.** By OTTILIE WALLACE. Pp. x., 52. Illustrated. London: Oxford University Press. 1931. Price 2s. 6d.—This small volume describes the experiences of one who was condemned for many months to the wearing of a caliper splint. The pitfalls which await such a patient are many and varied, and are oft-times such as would not be anticipated by the sound of limb. The author depicts these traps for the unwary with a vivid personal touch, and she has devised ingenious ways of avoiding and circumventing them. Her dodges for taking a bath or even getting into bed make quite amusing reading. Most doctors could pick up useful hints worth passing on to any patient who is learning to walk in a splint, while the book should prove a real boon to convalescing fracture cases.

